# The efficacy of a self-help parenting program for parents of children with externalizing behavior: a randomized controlled trial

**DOI:** 10.1007/s00787-022-02028-0

**Published:** 2022-07-07

**Authors:** Suzanne R. C. de Jong, Barbara J. van den Hoofdakker, Lianne van der Veen-Mulders, Betty Veenman, Jos W. R. Twisk, Jaap Oosterlaan, Marjolein Luman

**Affiliations:** 1 Department of Clinical‐, Neuro‐, and Developmental Psychology, Vrije Universiteit Amsterdam, Amsterdam, The Netherlands; 2 Department of Child and Adolescent Psychiatry, University Medical Center Groningen, University of Groningen, Groningen, The Netherlands; 3 Accare Child Study Center, Groningen, The Netherlands; 4 Department of Clinical Psychology and Experimental Psychopathology, University of Groningen, Groningen, The Netherlands; 5 Department of Epidemiology and Data Science, Amsterdam University Medical Centers, Amsterdam, the Netherlands; 6 Department of Pediatrics, Emma Neuroscience Group, Emma Children’s Hospital, Amsterdam UMC, University of Amsterdam, , Amsterdam Reproduction and Development Research Institute, Amsterdam, The Netherlands; 7 Levvel, Specialists in Youth and Family Care, Amsterdam, The Netherlands

**Keywords:** Behavioral parent training (BPT), Externalizing behavior problems, Prevention, Self-help parenting program

## Abstract

Parenting programs are effective for children with externalizing problems, but not always easily accessible for parents. In order to facilitate accessibility, we developed a self-help parenting program, consisting of a manual and online part. The efficacy of the program in reducing children’s externalizing problems was compared to waitlist in a randomized controlled trial. In addition, two versions of the program were exploratively compared, one with and one without biweekly telephonic support. Candidate moderators (child and parent factors) and parental satisfaction were also examined. We randomly assigned 110 families to one of the following three conditions: the support condition, the no support condition, or the waitlist condition. Intervention duration was 15 weeks. Outcomes were collected at baseline (T0), 8 weeks (T1), 15 weeks (T2), and 28 weeks (T3) and included daily telephonic measurements of parent-rated externalizing behavior and the Intensity scale of the parent-rated Eyberg Child Behavior Inventory (ECBI). Main analyses compared outcomes at T2, using longitudinal regressions with T0 as fixed factor. Results showed that children improved significantly more on both outcomes in the intervention condition compared to waitlist, with small to medium effect sizes. Parental satisfaction was high. Neither differences in efficacy nor in parental satisfaction were found between the support and no support condition. No moderators were detected. The newly developed self-help parenting program is effective in reducing children’s externalizing behavior problems and may help improve access to evidence-based care.

**Supplementary Information:**

The online version contains supplementary material available at 10.1007/s00787-022-02028-0.

## Introduction

Parenting programs are effective first-line treatments for children with externalizing behavior problems, such as hyperactive, impulsive and disobedient behavior, temper tantrums, and irritability [1, 2]. Research has shown that these programs reduce parent-rated symptoms of attention-deficit/hyperactivity disorder (ADHD) and oppositional defiant disorder (ODD) [3] with small to medium effect sizes [4, 5]. For ADHD symptoms, these effects are mixed when using masked assessments (for example, a home observation) [4–7], while for ODD symptoms, effects on parent-rated measures are corroborated by (probably) masked informants [8–10]. Parenting programs are designed to teach parents behavioral techniques to modify the child’s behavior and are usually delivered face-to-face (individually or in a group) by trained therapists [11] (e.g., [12]). However, parenting programs are often not easily accessible for parents, for example, due to a lack of qualified trainers, no availability of treatments nearby, or parental time constraints [13]. Low accessibility may lead to no or ineffective treatment, escalation of mild problem behavior, or unnecessary medication use [14, 15].

A promising way to improve the accessibility of parent training is by offering self-help programs [16]. Two meta-analyses showed that self-help parenting programs result in a small- to large-sized reduction of parent-reported externalizing child behavior [16, 17] and small to medium-sized improvement of parenting skills and parental well-being. These effects are comparable to the effects of face-to-face parenting programs [4, 5], but could not be confirmed in (a relatively scarce number of) studies using masked measures [16].

Self-help programs can be delivered with or without additional support, such as online feedback, reminders, or phone calls. Meta-analytic research on the effectiveness of such support shows conflicting evidence; one meta-analysis found some advantage of additional support consisting of either supportive phone calls or online feedback [16]. Another meta-analysis [17] found that programs that included reminders were more effective than programs without reminders, but that programs including telephonic coaching, were less effective than programs without this coaching. So far, no studies directly compared the effectiveness of a self-help program with and without additional support.

For the current study, we developed a Dutch self-help program for parents of children with externalizing behavior, using elements of a Dutch program available in both face-to-face and blended formats [12, 18], and a German self-help parenting program [19]. The 15-week self-help parenting program consists of a manual and online program. The primary aim of this study was to investigate the program’s efficacy in reducing parent-rated child behavioral problems, assessed with a questionnaire as well as through daily phone calls (which is considered a more ecologically valid method than commonly used questionnaires [20]). Efficacy was evaluated directly after the intervention and three months later. The secondary aim was to exploratively compare two versions of the program: a version with biweekly supportive telephone calls and a version without this additional support. Further, using parent-rated questionnaires, several possible moderating variables were explored, i.e., baseline severity of externalizing behavior [9], child’s age [8], sex and medication use, and parental age, sex and education level [21]. Finally, parents’ satisfaction was examined.

## Methods

### Design

We performed a non-masked, parallel, randomized controlled trial with three conditions: a) the support condition (self-help parenting program with telephonic support); b) the no support condition (self-help parenting program without telephonic support); and c) the waitlist condition. Main analyses combined both active conditions, from here on referred to as intervention condition. Allocation occurred by blocked randomization using three blocks (time periods) in which participants were pseudo-randomized to one of the three equally sized conditions. Randomization was performed by one of the authors (ML), who did not have contact with the participants, using a computer-generated random number list.

Participants in the intervention condition were instructed to complete the intervention in 15 weeks, while participants in the waitlist condition received the intervention after 15 weeks. Assessment took place at baseline (T0), 8 weeks (T1), 15 weeks (T2), and three months of follow-up (at 28 weeks, T3). T3 measurements were omitted for participants in the waitlist condition.

### Participants

Participants were parents seeking support for their child’s externalizing behavior. Recruitment took place through local child support facilities, social media, schools, and a Dutch parent association for children with developmental problems.

**Fig. 1:**
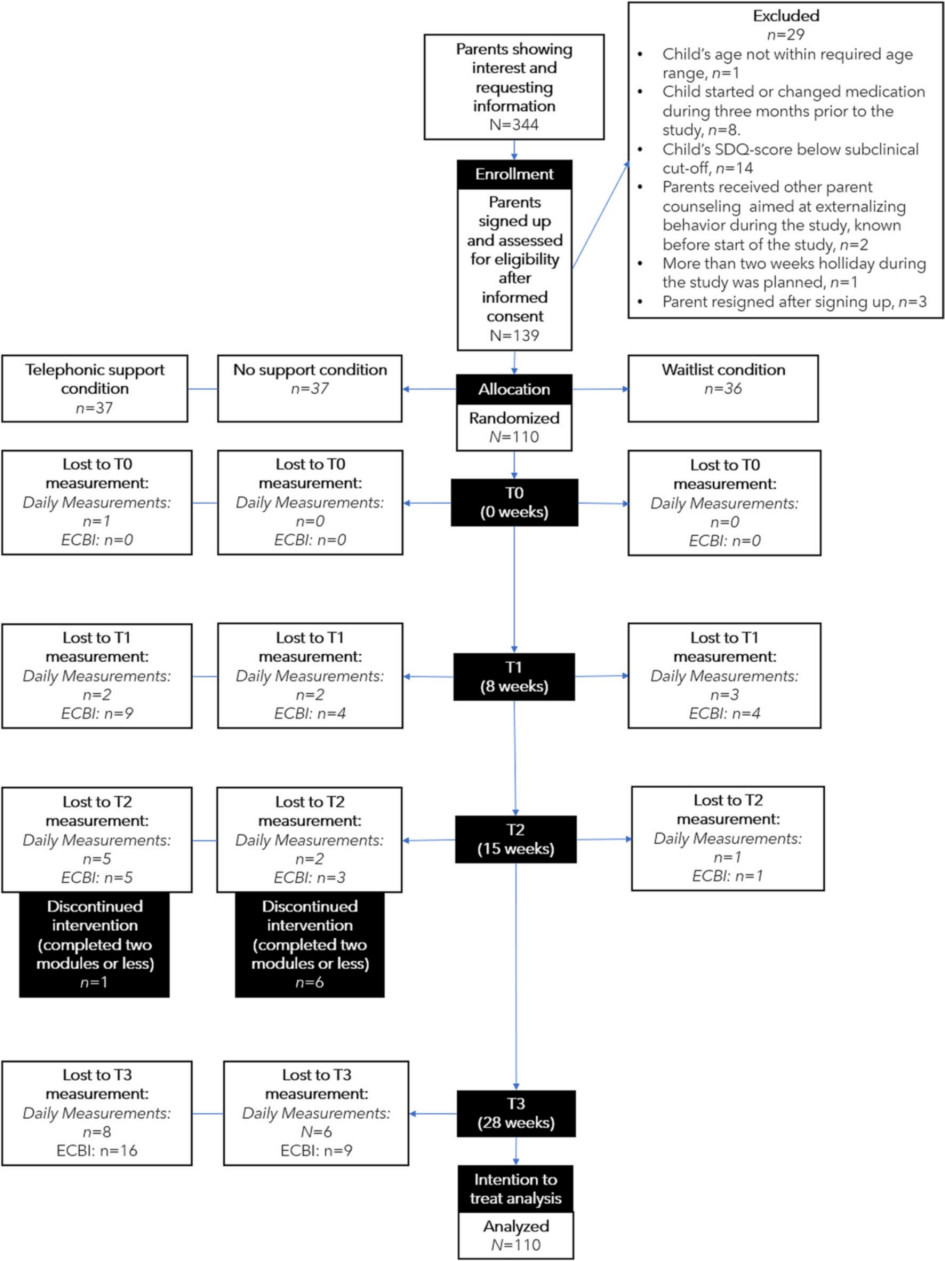
Flowchart (According to CONSORT Statement; [27]). Notes: *ECBI* Eyberg Child and Behavior Inventory; *SDQ* Strengths and Difficulties Questionnaire

Parents were included if (1) they were voluntarily seeking help for their child’s externalizing behavior; (2) their child was between 4 and 12 years old; (3) their child showed (sub)clinical levels of externalizing behavior at home, as indicated by a) a score above the 80th percentile (i.e., score > 6 for children aged 4 or 5 years and > 7 for children aged 6–12) on the externalizing behavior scale of the Strengths and Difficulties Questionnaire (SDQ; [22]) and b) displaying at least three ADHD symptoms (either hyperactivity/impulsivity and/or inattention) and/or at least two ODD symptoms on the Diagnostic Interview Schedule for Children fourth edition (DISC-IV; [3, 23, 24]); and (4) their child suffered from impairment due to externalizing behavior, as indicated by scoring ≥ 4 on one of the domains of the Impairment Rating Scale (IRS; [25]). Parents were excluded if (1) they were receiving other parent training during this study or in the preceding six months; (2) they received other parent counseling, directed at externalizing behavior of the child at home, during this study; (3) their child started psychotropic medication or changed medication dose up to three months prior to this study; (4) their Dutch reading ability was not sufficient to understand the program materials; and (5) they went on holiday during this study for more than 2 weeks. Figure 1 displays the flow chart of participant inclusion.

 A power analysis (see [26]) for our main analyses comparing the intervention to waitlist, based on two measurements (T1 and T2), two groups with a two-to-one ratio, and a medium effect size (0.5; in line with effect sizes in comparable studies [16, 17]) resulted in a required sample size of 113.

## Materials

### Intervention

The self-help parenting program concerns a 15-week program, consisting of a manual and online part. It offers some psycho-education and provides parents with behavioral parenting techniques to modify their child’s externalizing behavior, such as providing structure, praise appropriate behavior, ignoring unwanted behavior, and applying mild punishment [11, 28]. The program consists of 11 modules (see supplementary materials A). Each module requires parents to read a chapter of the manual, providing information about a particular technique. Using the online program, parents are quizzed about the technique, make a plan to implement the technique, and monitor their child’s behavior and their parenting confidence. Parents were encouraged to use both the manual and online program, by referring from the manual to the online program and vice versa. Parents start the program by choosing three to five behaviors which they would like to see improved, based on frequency and severity, which they work with and are monitored during the program. Some parts of the program, for example, a module about temper tantrums, are only offered if the child shows the particular behavior targeted in that part.

Two versions of the program were developed: one without additional support and one with biweekly protocolized, telephonic support (see supplementary materials B). The goal of these calls was to motivate parents, monitor progress, and answer questions, without introducing new knowledge. Furthermore, for some specific techniques it was discussed whether parents used these techniques correctly and if necessary, parents were provided with advice, using information from the manual. In case parents were more than two weeks behind schedule or indicated the desire to quit, they were motivated to continue. The phone calls were carried out by master level psychologists. The phone calls were recorded and ten percent of them were checked for completeness (in percentages) and protocol fidelity (0 = no protocol violation, 1 = one minor protocol violation, 2 = one major protocol violation or multiple minor violations) by two of the authors (ML and BvdH). A protocol violation may be that advice is given outside the information provided in the program, or failing to answer a parent’s question according to protocol.

### Measurements

#### Screening

Screening for externalizing behavior was done using the Externalizing scale of the Strengths and Difficulties Questionnaire (SDQ) [22], consisting of five ADHD and five behavior disorder-related symptoms, that parents evaluate by indicating how often their child shows this behavior on a three-point Likert scale (0 = not true, 2 = certainly true). The validity of the total SDQ was shown to be good and the reliability of the externalizing scale is considered acceptable [29, 30].

To screen for ADHD and ODD symptoms, the ADHD and ODD part of the structured Diagnostic Interview Schedule for Children (DISC [24]) were administered to parents by telephone, in which parents are asked to indicate if a symptom was present during the last six months. Psychometric properties of the ADHD and ODD sections of the DISC are moderate to excellent [31, 32].

The parent-rated Impairment Rating Scale (IRS [25]) was used to assess impairment due to externalizing behavior problems on several aspects of the child’s life, such as the relationship with parents or siblings. The IRS is scored on eight 11-point Likert scaled items (0 = no impairment, 10 = extreme impairment). Psychometric properties are considered good [25].

#### Outcome measures

Daily externalizing behavior was assessed using an Ecological Momentary Assessment (EMA) method. EMA is considered ecologically valid, minimizing recall bias, and is taking into account fluctuations in behavior over time [20]. During 3-min phone calls on four consecutive school days, parents were asked to indicate whether their child showed specific externalizing behavior in the past 24 h (for example “showed disobedience” or “was hyperactive or restless”). In case a particular behavior occurred, parents indicated the severity of this behavior on a five-point scale (1 = not severe, 5 = very severe). The behaviors (14 in total) were selected from an adapted version of a list of target behaviors (see [12]) and were identical to the behaviors that could be targeted during the intervention. The short phone calls were protocolized and conducted by trained research assistants, who were unaware of study condition. The mean of the four-day scores of all the items was used as outcome (daily measurements). If parents missed two or more measurements, measurements were taken the following week. If taking measurements also did not succeed the next week, they were considered missing. Parents who had missing data for three or more days were excluded from that measurement week. When parents reported the child being ill or that they (almost) did not see the child on a particular day, the measurement of that day was omitted. Internal consistency during baseline in the current sample was averaged for all measurement days and showed good reliability (*α* = 0.83).

Externalizing behavior was further assessed with the parent-reported Eyberg Child Behavior Inventory, Intensity scale (ECBI [33]). This scale contains 36 items stating a certain problem behavior, of which frequency is indicated on a seven-point Likert scale (1 = never, 7 = always). Psychometric properties of the Intensity scale are good. Previous research demonstrated excellent internal consistency (*α* = 0.92), good test–retest reliability (*α* = 0.84) and good convergent, divergent, and discriminative validity [34]. Reliability in the present study was good (*α* = 0.81).

To assess parents’ satisfaction with the program, an adaptation of the Client Satisfaction Questionnaire (CSQ-8 [35]) was used. The CSQ-8 consists of eight questions on four-point Likert scales, with higher scores indicating greater satisfaction. Psychometric properties are good [36]. Furthermore, parents were asked an overall evaluation of the program on a scale from 1 (very dissatisfied) to 10 (very satisfied).

Demographic characteristics (i.e., child’s age, sex, and parent’s age, sex and educational level) and child’s medication use at baseline were assessed using a custom-made questionnaire (using guidelines for reporting educational level [37]). Likewise, a custom-made questionnaire was used for assessing consumption of (additional) mental health care regarding the child’s behavioral problems and medication switch between T0 to T2 and T2 to T3.

### Procedure

This study was conducted between February 2019 and March 2021. Parents could start this study at any week, except for the weeks in which measurement weeks would take place during school holidays. However, during the COVID-19-related school closings, participants were allowed to start any week, since the differences between school and holiday weeks were small during that time. Parents could participate either alone or together with their partner, if being part of the same household. The parent most involved in the intervention was considered the primary parent and filled out the questionnaires (or optionally together with their partner, if being part of the same household). After commencing this study, all participants were allowed to seek and receive (additional) care.

Medical ethical approval of this study was waived for the medical research with human subjects act [38] by the Medical Ethical Committee of the VU Medical Centre (#2018.421). The trial was registered in the *Netherlands Trial Register* (https://www.trialregister.nl/trial/8200). CONSORT guidelines were followed to describe this study [27].

### Data analysis

Data were analyzed based on intention-to-treat. Statistical analyses were performed using the Statistical Package for Social Sciences (SPSS [39]) and STATA [40]. Outliers were detected by boxplots and winsorized [41] when they exceeded three standard deviations from the mean (which showed to be a maximum of one case per measure). Possible differences between conditions regarding child and parent demographic characteristics were assessed with t-tests (two conditions) or ANOVA’s (three conditions) for continuous/ordinal data or Fisher’s exact tests for categorical data. Furthermore, during T0–T2 it was checked whether the amount of additional care received (including medication start or stop) did not significantly differ between the conditions. Likewise, it was checked whether the number of parents participating in the parenting program and the number of parents that had a measurement week during the COVID-19-related school closing did not significantly differ between conditions. Furthermore, the number of modules that parents worked with in the online program was compared between the support and the no support condition. If parents entered at least one response in a particular module in the online program, we assumed that parents had worked with that particular module. Inter-rater reliability of the ratings of the two authors for completeness and protocol fidelity of the supportive phone calls was assessed using B statistic, since nominal and ordinal categories were used [42].

Efficacy of the self-help program was analyzed by comparing (in separate analyses) Daily Measurements and ECBI scores in the intervention versus waitlist condition, using longitudinal mixed-model regression analysis. There were two hierarchical levels: observations (level 1) were nested in participants (level 2). Group was added as between subject variable and time (T1–T2) as within subject variable. Main outcome was the difference between groups at T2. To adjust for possible baseline differences in outcome measures between conditions, baseline measurement (T0) was added as fixed factor in the model [43]. Effect sizes of group differences were calculated by dividing the mean differences by the pooled standard deviation of the outcome (*Cohen’s d* [44]).

To exploratively assess differences between the support and no support condition, longitudinal mixed-model analysis was repeated with three conditions for both Daily Measurements and ECBI, also including T3. To test whether Daily Measurement and ECBI scores changed between T2 and T3, two separate within group regressions were done in the support and the no support condition. To exploratively assess moderators, candidate variables (i.e., child symptom level, age, sex, and medication use at baseline, and parent age, sex, and education level) were added as a condition*moderator interaction to the two-condition models. In case of a significant interaction, post hoc tests were carried out within each categorical group or within three equally sized groups for continuous data. Lastly, the difference in parental satisfaction (CSQ and general satisfaction) between the support and no support condition was analyzed with *t*-tests.

Because we had two primary outcome measures, the significance level for the analyses regarding ECBI and daily measurements was set at *α* = 0.025 (Bonferroni correction [45];). We used *α* = 0.05 for the moderator analyses and analyses regarding satisfaction and possible confounders.

## Results

### Participants

Children had a mean age of 8.17 years (SD = 2.29) and 80 of them were boys (72.7%). Primary parents had a mean age of 40.56 years (SD = 5.31) and 101 (91.8%) of them were female. Baseline demographic variables of the intervention and waitlist condition are shown in Table 1. Baseline variables (i.e., child symptom level, age, sex, impairment, and medication use, and parent age, sex, education level, and household composition) did not significantly differ between the two conditions (nor between the three conditions, see Table S1, supplementary materials C).

**Table 1:** Baseline child and parent demographic characteristics in the intervention and waitlist condition, and comparisons between the two conditions

	Intervention condition (*n* = 74)	Waitlist condition (*n* = 36)	Condition comparisons
Baseline child characteristics			
Age in years, *M* (SD)	8.24 (2.32)	8.14 (2.26)	*t* (108) = − 0.22, *p* = 0.829
Sex: boys, *n* (%)	50 (67.6)	30 (83.3)	Fisher’s exact: *p* = 0.110
SDQ, *M* (SD)^a^	12.01 (2.24)	11.14 (2.17)	*t* (107) = − 1.94, *p* = 0.054
ADHD (DISC-IV), *n* (%)			Fisher’s exact: *p* = 0.352
Clinical	55 (74.3)	31 (86.1)	
Subclinical	18 (24.3)	5 (13.9)	
Non-clinical	1 (1.4)	0 (0)	
ODD (DISC-IV), *n* (%)			Fisher’s exact: *p* = 0.403
Clinical	57 (77.0)	31 (86.1)	
Subclinical	14 (18.9)	3 (8.3)	
Non-clinical	3 (4.1)	2 (5.6)	
Impairment, *M* (SD)^b^	6.23 (1.56)	5.93 (1.38)	*t* (108) = − 0.25, *p* = 0.579
Psychotropic medication, *n* (%)^c^	16 (21.6)	12 (33.3)	Fisher’s exact: *p* = 0.244
Primary parent characteristics			
Age in years, *M* (SD)	40.68 (4.88)	40.32 (6.21)	*t* (101) = − 0.32*, p* = 0.747
Sex: females, *n* (%)	68 (91.9)	33 (91.7)	Fisher’s exact: *p* = 1.000
Education level, *M (SD)*^d^	5.15 (1.04)	4.88 (1.07)	*t* (103) = − 1.25,* p* = 0.215
Household composition: single parent, *n* (%)	8 (10.8)	2 (5.6)	Fisher’s exact: *p* = 0.493

*ADHD* attention-deficit/hyperactivity disorder, *DISC-IV* Diagnostic Interview Schedule for Children fourth edition, *ODD* oppositional defiant disorder, *SDQ* Strength and Difficulties Questionnaire

^a^Range: 0–10

^b^Range: 0–20

^c^Intervention condition: 13 methylphenidate, 1 dexamphetamine, 1 lisdexamfetamine, 1 aripiprazole; Waitlist condition: 12 methylphenidate

^d^Percentage of parents with no education or primary education: 1.9%; lower or upper secondary education: 26.7%; (under)graduate or postgraduate: 71.4%

There were no significant differences between conditions on any of the possible confounding variables between T0 and T2: amount of additional care (including medication start or stop) and number of parents participating during the COVID-19-related school closing (see Table S2, supplementary materials D). Furthermore, there was no significant difference in the number of online modules that parents worked with between the support condition (*M* = 7.30, SD = 2.22) and the no support condition, although a trend toward more modules in the support condition was found (*M* = 6.11, SD = 3.23), *t* (63.84) = 1.85, *p* = 0.070.

The mean number of phone calls per parent(s) was 6.11 (SD = 1.74) and the average length of the phone calls was 12 min. Inter-rater reliability was good for both covering the required protocol topics (*B* statistic = 0.88) and providing answers according to protocol (*B* statistic = 0.67). Protocol fidelity score was 95 percent for covering the required protocol topics. Regarding providing protocolized answers, in 70 percent of the phone calls there were no protocol violations (score 0), in 30 percent there was one minor violation (score 1), and there were no phone calls with major or multiple protocol violations (score 2).

### Comparison between intervention and waitlist

**Fig. 2:**
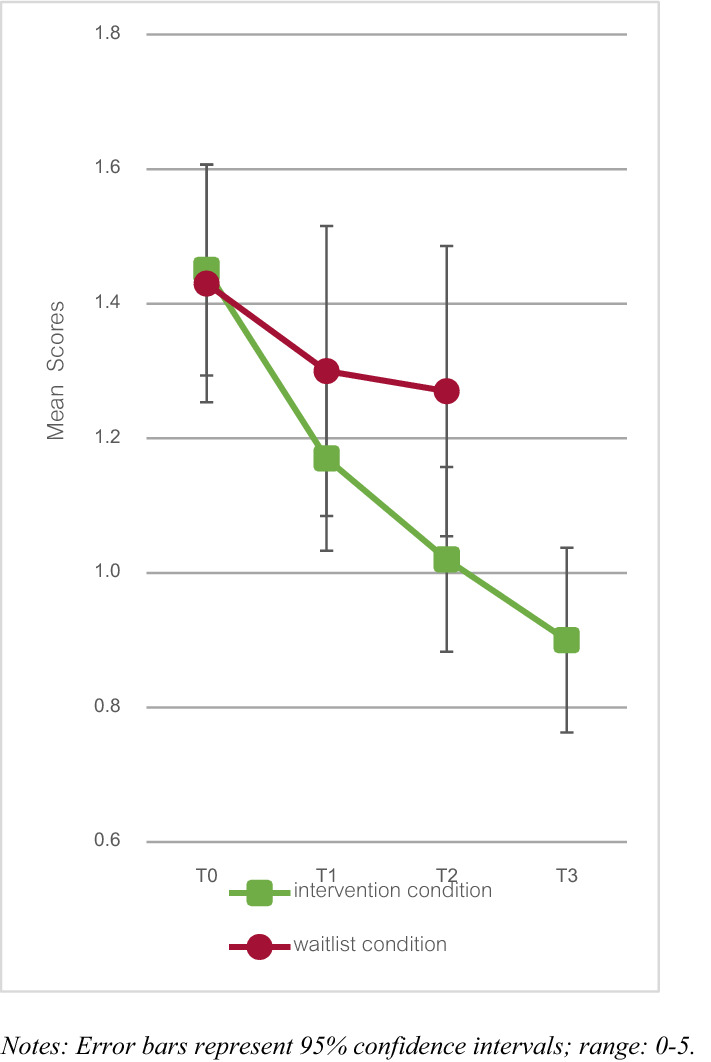
Daily measurements scores for the intervention and waitlist condition

Results regarding the Daily Measurements are displayed in Fig. 2. At T2, a significantly lower Daily Measurements score was found for the intervention condition compared to the waitlist condition, (*B *(SE) = − 0.27 (0.11), *p* = 0.011), with a small effect size (*d* = − 0.43). Regarding long-term effects, within the intervention condition, no significant change was found between T2 and T3 (*B* (SE) = − 0.11 (0.07), *p* = 0.125), indicating persistence of changes.

**Fig. 3:**
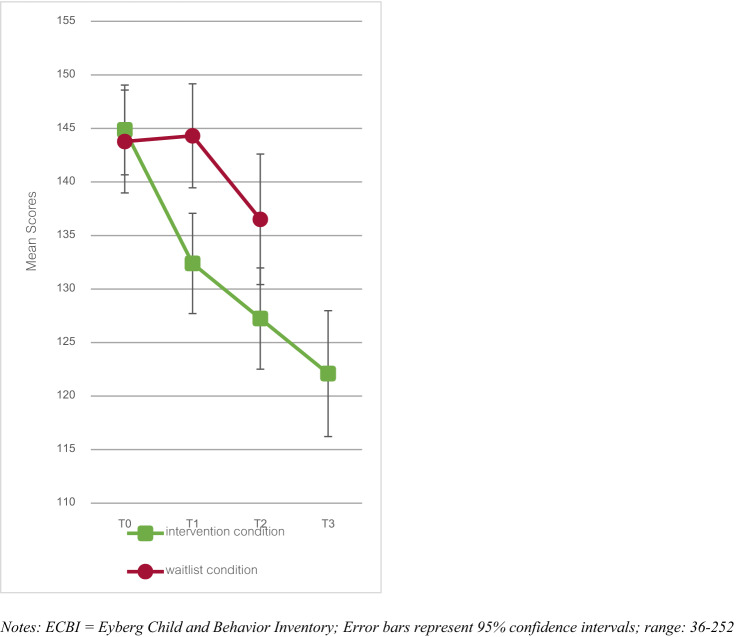
ECBI scores for the intervention and waitlist condition

Results regarding the ECBI are displayed in Fig. 3. At T2, a significantly lower ECBI score was found in the intervention condition compared to the waitlist condition, (*B* (SE) = − 9.81 (2.61), *p* < 0.001) with a medium effect size (*d* = − 0.51). Regarding long-term effects, within the intervention condition, no significant change was found between T2 and T3 (*B* (SE) = − 3.48 (1.81), *p* = 0.055), indicating persistence of changes.

### Exploratory analyses

**Fig. 4:**
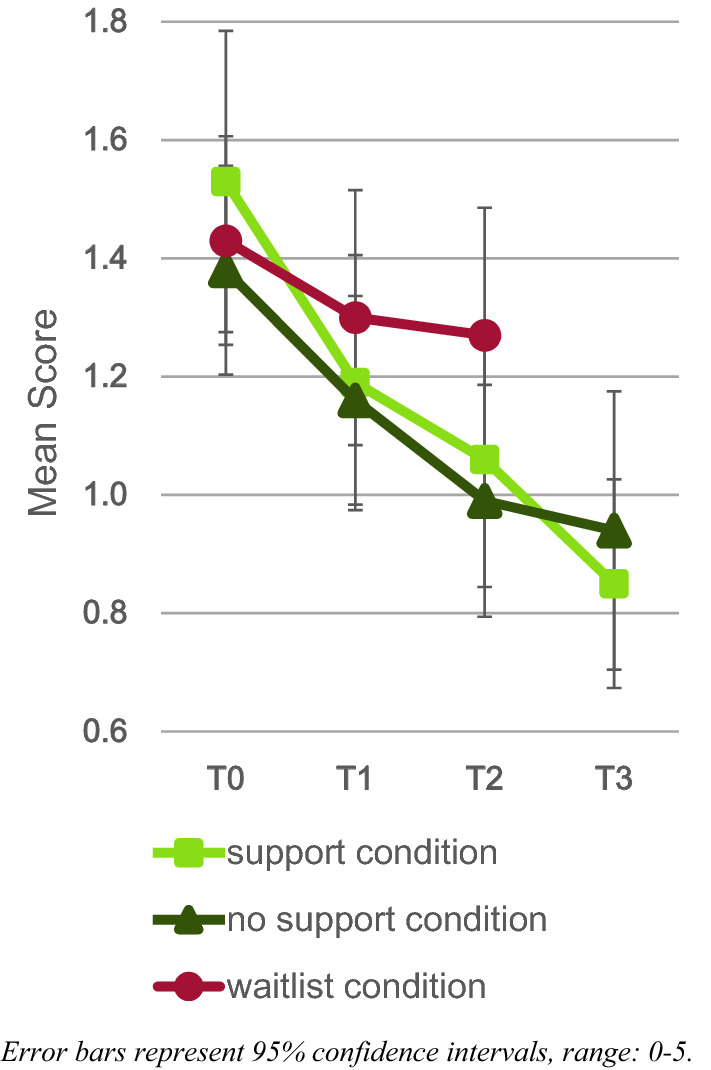
Daily measurements scores for the support, no support, and waitlist condition

**Fig. 5:**
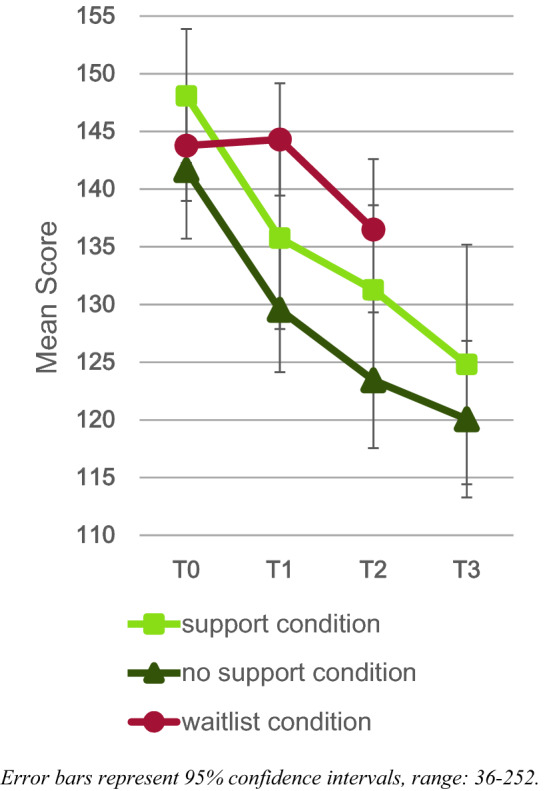
ECBI scores for the support, no support, and waitlist condition

Results regarding the support versus no support condition comparisons are displayed in Tables S3 and S4 (supplementary materials E) and Figs. 4 and 5. Findings showed that there were no significant differences between the support condition and the no support condition at T2 or at T3. Within-group analyses revealed that, from T2 to T3, there was a significant decline in ECBI in the support condition only.

Moderator analyses showed that neither child and parent characteristics (i.e., child age, sex, and medication use, and parent age, sex, and education level) nor baseline levels of daily measurements and ECBI significantly moderated the differences between the intervention and waitlist condition (*p* > 0.056).

### Program satisfaction and consumption of mental health care

Mean CSQ score was 3.20 (SD = 0.51) out of 4 and parents valued the parenting program on average with 7.90 (SD = 1.24) out of 10. There were no significant differences in parental satisfaction between the intervention conditions (CSQ: *p* = 0.299, overall evaluation: *p* = 0.214). Between T2 and T3 (after the intervention), 79.4 percent of the children and/or parents in the intervention condition who filled out the questionnaire (*n* = 45, 61%) did not receive additional mental health care for child behavioral problems.

## Discussion 

The present study showed that our newly developed self-help parenting program was efficacious in reducing child externalizing behavior compared to waitlist, with small to medium effect sizes. The behavior change observed directly after the program did not diminish up to three months later, suggesting perseverance of intervention gains. No differences were found between the program formats with and without additional support and no moderators of efficacy were detected.

The observed reductions in externalizing behavior are in line with previous research [16, 17]. Also in line with previous research was the persistence of changes three months after the intervention period [16], which might show that parents kept using the techniques after the intervention. In addition to previous research, we used an ecologically valid measurement and had three measurement points, which added to the reliability and validity of the results.

As a proxy for clinical significance, we checked whether children and/or parents received mental health care related to the child’s externalizing behavior after the intervention. It was found that of those families who received the self-help program, 79.4 percent of children and parents did not receive additional care in the three months after the intervention. This may suggest that the majority of the children improved to a level for which the use of mental health care w as no longer necessary. However, this finding should be interpreted with caution, because other explanations for not making use of further treatment may be possible, such as not wanting to seek further treatment immediately after receiving an intervention, time constraints, or lack of confidence in further treatment. Furthermore, only 61 percent of the parents who received the intervention filled out the follow-up questionnaire. Moreover, we could not compare mental health care consumption of families that followed the intervention to those assigned to waitlist, because participants on the waitlist received the parenting program between T2 and T3, and therefore did not fill out the follow-up questionnaire. Future research should take into account other measures to assess the clinical significance of findings, such as functional impairment.

Our study was the first to exploratively compare a self-help parenting program with and without additional support and found no differences in efficacy, nor in parental satisfaction. Previous meta-analyses on the difference in effectiveness of programs with and without additional support were mixed. One meta-analysis showed a minor advantage for programs with additional support (either phone calls or online feedback) [16], while another found that programs with telephonic support were overall less effective than programs without this element. However, only taking the presence versus absence of this particular element within different parenting programs into account could have led to confounding factors influencing results, if the particular element was not the only shared element [17]. The absence of differences between the two formats might have an important clinical implication, as without additional support, the accessibility of the program increases. However, the comparison between the format with and without additional support was exploratory, and should be interpreted with caution, as our sample size may have been insufficient for this head-to-head comparison. Moreover, the mixed findings in existing literature regarding additional support might be due to a great variability in the kind of additional support (e.g., telephonic or online), as well as the frequency and the intensity of the offered support (e.g., written feedback or intense coaching sessions by telephone). Therefore, the optimal form of this support awaits further study.

Parent and child factors (child’s age, sex, and medication use, and parental age, sex, and education level) and baseline problem severity did not moderate efficacy. Efforts were made to include a diverse group of parents in terms of SES and migration background, by recruiting participants via several channels, putting effort in reaching less accessible groups, and using clear and concise written information on this study. However, the education level of parents in our sample was relatively high (71 percent had followed higher education, versus an average of 30 percent in the Netherlands [46]. Moreover, parental education level tends to be lower in samples of children with externalizing behavior problems [47]). Furthermore, most parents in our sample had a non-migration background. Most previous research on self-help programs included similar samples [48–50], which might suggest that parents with a migration background or lower education level face difficulties finding their way to parenting programs. It is known that in general these parents have difficulties reaching mental health care for themselves or their children [51–53].

In terms of severity of behavior, our sample showed relatively high (clinical) levels of externalizing behavior and few children with low levels of externalizing behavior. This might explain that we did not find severity of problem behavior moderating efficacy, while other studies found larger efficacy of parenting programs when taking more severely disturbed children into account [9]. Although future studies may investigate the efficacy of self-help programs in a more diverse population and with a larger sample, the absence of moderators in the present study may suggest the program to benefit a broad population of children.

Finally, the current study relied solely on parent-reported outcomes. Obviously, parents were not masked for study condition and put substantial effort into the intervention, which may have led to overestimation of the intervention effects [7, 54]. It could be argued that when measuring the effects of an intervention for child externalizing behavior, a masked measure should be included in order to increase validity (e.g., [7]). However, the perception of parents might be more relevant than an objective measure of the child’s behavior. To increase validity within the current study, we used an EMA measure taken by masked assessors, which is more ecologically valid and less susceptible for recall bias than commonly used questionnaires [20].

## Limitations and further research

Some limitations of our study should be taken into account. First, since power calculations were based on expected medium-sized differences in efficacy between the intervention versus waitlist, and a potential difference between the two formats of the parenting program might be small, power might have been insufficient for that comparison. Likewise, power was an issue for the moderator analyses. Future research should replicate the results with a larger sample and investigate whether there might be subgroups of parents who benefit more from the program and/or benefit more from additional support. Second, in both parenting program versions, parents received progress questionnaires. Sending out these questionnaires might have stimulated parents to continue their participation in the program [17]. Without these reminders, efficacy of the two formats might have been meaningfully different. Third, results of the follow-up measurements of the ECBI should be interpreted with caution, since a relatively high proportion of data were missing. Finally, data were partly collected during the COVID-19-related school closing. This could have led to more variability in the data, because homeschooling could reduce or provoke (other) externalizing behavior. However, this unlikely impacted our findings, because the number of families participating during the COVID-19-related school closing did not differ between the conditions.

## Implications/conclusion

The present study showed small- to medium-sized efficacy of our self-help parenting program aimed at reducing children’s externalizing behavior problems. The intervention is aligned with clinical guidelines, which advises parent training as first-line treatment for externalizing behavior [1, 2]. Our findings suggest that parents can use the program without additional support, although this needs to be confirmed in a larger sample. Our program can increase access to evidence-based care for parents and their children and it might safe costly child care resources. Further research should study if the program indeed prevents referral to more intensive interventions.

### Supplementary Information

Below is the link to the electronic supplementary material.

Supplementary file1 (DOCX 27 kb)
