# Supplementary material for: The efficacy of a self-help parenting program for parents of children with externalizing behavior: a randomized controlled trial
Source: Eur Child Adolesc Psychiatry. 2022 Jul 7;32(10):2031–42. doi: 10.1007/s00787-022-02028-0 (PMC9261243; doi:10.1007/s00787-022-02028-0)
Supplement: Supplementary file 1 — Supplementary file1 (DOCX 27 kb) [file 787_2022_2028_MOESM1_ESM.docx]

**Supplementary Materials**

**The Efficacy of a Self-help Parenting Program for Parents of Children with Externalizing Behavior: a Randomized Controlled Trial**

Suzanne R.C. de Jong^1^, Barbara J. van den Hoofdakker ^2,3,4^, Lianne van der Veen-Mulders ^2^, Betty Veenman ^3^, Jos W.R. Twisk ^5^, Jaap Oosterlaan^1,6^, Marjolein Luman ^1,7^

*^1^: Department of Clinical‐, Neuro‐, and Developmental Psychology, Vrije Universiteit Amsterdam, Amsterdam, The Netherlands*

*^2^: Department of Child and Adolescent Psychiatry, University Medical Center Groningen, University of Groningen, Groningen, The Netherlands*

*^3^: Accare, University Centre for Child and Adolescent Psychiatry Groningen, The Netherlands*

*^4^: Department of Clinical Psychology and Experimental Psychopathology, University of Groningen, Groningen, The Netherlands*

*^5^: Department of Epidemiology and Data Science, Amsterdam University Medical Centers, Amsterdam, the Netherlands*

*^6^: Emma Children's Hospital, Amsterdam UMC, University of Amsterdam, Department of Pediatrics, Emma Neuroscience Group, Amsterdam Reproduction & Development research institute, Amsterdam, The Netherlands*

*7. Levvel, specialists in youth and family care, Amsterdam, The Netherlands*

**Corresponding author:**Suzanne R.C. de Jong
[s.r.c.dejong@vu.nl](mailto:s.r.c.dejong@vu.nl)

**A: Overview of the Self-Help Parenting Program Modules**

Module 1: Introduction

Module 2: Disruptive behavior

Module 3: Providing positive attention

Module 4: Playing with your child

Module 5: Providing structure

Module 6: Rules and instructions

Module 7: Extra reinforcement

Module 8: Ignore unwanted behavior

Module 9: What to do with unacceptable behavior?

Module 10: Techniques regarding temper tantrums

Module 11: Relapse prevention and disruptive behavior at school

**B : Main Questions Asked During the Protocolized Telephone Calls**

- Which modules did you work with the past the past two weeks?
- Did you manage to complete reading the manual and to complete the online program for these modules?
- Did you manage to implement the techniques? (if applicable)
- Do you have any questions about these modules?
- Do you have any other question
- Specific questions about the techniques, only for specific modules:
  - Module 1 : Which behavior did you choose to work with?
  - Module 3: How often do you praise your child on a regular day?
  - Module 6: Can you give an example of a rule you applied and of an instruction you gave your child?
  - Module 8: Which behavior did you choose to apply the techniques regarding ignoring?
  - Module 9: Which behavior did you choose to apply the techniques regarding consequences for unwanted behavior?
- If applicable: asking why the parent did not continue the program and making a plan to regain motivation and progress.

Maximum length of the phone calls was 30 minutes.

**C: Baseline Characteristics**

**Table S1** *Baseline Child and Parent Demographic Characteristics in the Support, No Support and Waitlist Condition, and Comparisons Between the Three Conditions*

|  | Support  condition  (N=37) | No support  condition  (N=37) | Waitlist  condition  (N=36) | Three condition comparisons |
| --- | --- | --- | --- | --- |
| *Child characteristics* |  |  |  |  |
| Age in years, *M (SD)* | 7.95 (2.37) | 8.53 (2.26) | 8.14 (2.26) | *F* (2, 107) = 0.62, *p* = .539 |
| Sex: boys, *N* (%) | 26 (70.3) | 24 (64.9) | 30 (83.3) | *Fishers’s exact: p* = .208 |
| SDQ, *M (SD)*^a^ |  |  |  | *F* (2, 107) = 2.04, *p* = .135 |
| ADHD (DISC-IV), *N* (%)  Clinical  Subclinical  No classification | 26 (70.3)  10 (27.0)  1 (2.7) | 29 (78.4)  8 (21.6)  0 (0) | 31 (86.1)  5 (13.9)  0 (0) | *Fishers’s exact: p* = .358 |
| ODD (DISC-IV), *N* (%) |  |  |  | *Fishers’s exact: p* = .387 |
| Clinical | 31(83.8) | 26 (70.3) | 31 (86.1) |  |
| Subclinical | 5(13.5) | 9 (24.3) | 3 (8.3) |  |
| No classification | 1(2.7) | 2 (5.4) | 2 (5.6) |  |
| Impairment *M (SD)*^b^ | 6.23 (1.56) | 5.93 (1.38) | 6.01 (1.28) | *F* (2,107) = 0.47, *p* = .510 |
| Psychotropic medication^c^, *N* (%)^a^ | 8 (21.6) | 8 (21.6) | 12 (33.3) | *Fishers’s exact: p* = .425 |
| *Primary Parent characteristics* |  |  |  |  |
| Age, parent *M (SD)* | 40.22 (5.12) | 41.12 (4.67) | 40.32 (6.21) | *F* (2, 100) = 0.30, *p* = .740 |
| Sex: females, *N* (%) | 35 (94.6) | 33 (89.2) | 33 (91.7) | *Fishers’s exact: p* = .768 |
| Education Level, *M (SD)* ^d^ | 5.26 (0.91) | 5.06 (1.15) | 4.88 (1.07) | *F* (2, 102) = 1.11, *p* = .335 |
| Household composition: single parent, *N* (%) | 3 (8.1) | 5 (13.5) | 2 (5.6) | *Fishers’s exact: p* = .599 |

*Notes: ADHD = attention deficit hyperactivity disorder, DISC-IV= Diagnostic Interview Schedule for Children fourth edition, ODD= oppositional defiant disorder, SDQ = Strengths and Difficulties Questionnaire ^a^range: 0-10 ^b^range: 0-20 ^c^support condition: 5* methylphenidate, 1 dexamphetamine, 1 lisdexamphetamine, , 1 aripiprazole, ; *no support condition:* 8 methylphenidate; *WL:* 12 methylphenidate;  *^d^percentage of parents with no education or primary education 1.9% ; lower or upper secondary education: 26.7%,; (under)graduate or post graduate: 71.4%.*

**D: Possible Confounders**

**Table S2** *Study Characteristics of the Participants in the Support, No Support and Waitlist Condition and Comparisons Between Intervention versus Waitlist Condition and Support versus No Support Condition*

|  | Support  Condition  (N=37) | No  support  Condition  (N=37) | Waitlist  Condition  (N=36) | Comparison intervention  versus  waitlistcondition | Comparison Support  versus  no support  condition |
| --- | --- | --- | --- | --- | --- |
| Other care regarding child’s externalizing behavior between T0-T2: N, (%) | 4 (12.1) | 3 (9.7) | 7 (20.0) | *Fishers’s exact: p*=.239 | *Fishers’s exact: p* =.515 |
| Switch medication between T0-T2 Started: N (%) | 2(5.4) | 2 (5.4) | 0 (0) | *Fishers’s exact: p* = .169 | *Fishers’s exact: p* =.543 |
| Stopped: N (%) | 0 (0) | 0 (0) | 1 (2.8) |  |  |
| Parenting program (partly) during Covid lockdown (schools closed): N (%) | 20 (54.1) | 21 (56.8) | - | - | *Fishers’s exact: p* *=*1.000 |
| Covid Lockdown (schools closed) at T0: N (%) | 2 (5.4) | 5 (13.5) | 4 (11.1) | *Fishers’s exact: p*=.748 | *Fishers’s exact: p* =.533 |
| At T2: N (%) | 3 (8.1) | 5 (13.5) | 4 (11.1) | *Fishers’s exact: p*=1.000 | *Fishers’s exact: p* =.810 |
| At T3: N (%) | 5 (13.5) | 4 (10.8) | - | - | *Fishers’s exact: p* = 1.000 |

**E: Three condition comparisons**

**Table S3** *Effects of cCondition Support versus No Support) at T2 and T3*

|  | T2 | | | T3 | | |
| --- | --- | --- | --- | --- | --- | --- |
|  | *B (SE)* | *p* | *d* | *B (SE)* | *p* | *d* |
| Daily Measurements^a^ | -0.02 (0.12) | .887 | -0.03 | -0.19 (0.13) | .121 | -0.32 |
| ECBI^b^ | 2.36 (3.08) | .442 | 0.12 | -1.08 (3.48) | .756 | -0.05 |

*Note: ECBI = Eyberg Child and Behavior Inventory, ^a^range: 0-5, ^b^range = 36-252*

**Table S4** *Within Group Effects of the Support and No Support Condition fromT2toT3*

|  | | T2- T3 | |
| --- | --- | --- | --- |
|  |  | *B (SE)* | *p* |
| Daily Measurements^a^ | Support condition | -.20 (0.11) | .072 |
|  | No support condition | -.02 (0.08) | .808 |
| ECBI^b^ | Support condition | -5.24 (2.49) | .035 |
|  | No support condition | -2.07 (2.58) | .423 |

*Note: ECBI = Eyberg Child and Behavior Inventory, ^a^range: 0-5, ^b^range = 36-252*
